# A Genetic Test of Sexual Size Dimorphism in Pre-Emergent Chinook Salmon

**DOI:** 10.1371/journal.pone.0078421

**Published:** 2013-10-21

**Authors:** Tosh W. Mizzau, Shawn R. Garner, Stephen A. C. Marklevitz, Graham J. Thompson, Yolanda E. Morbey

**Affiliations:** Department of Biology, Western University, London, Ontario, Canada; George Washington University, United States of America

## Abstract

Sex differences in early development may play an important role in the expression of sexual size dimorphism at the adult stage. To test whether sexual size dimorphism is present in pre-emergent chinook salmon (*Oncorhynchus tshawytscha*), alevins were reared at two temperatures (10 °C and 15 °C) and sexed using the *OtY1* marker on the Y-chromosome. Linear mixed models were used to test for sex differences in alevin size within families while controlling for the random effects of sire and dam nested within sire. Males and females did not differ in weight at 10 °C but males were heavier than females at 15 °C. Sex accounted for 2% of the within-family variance in weight. In addition, at 15°C, the relationship between weight and sex was greater in families with larger eggs. Whereas male-biased sexual size dimorphism was present at the juvenile stage, female-biased sexual size dimorphism was present at sexual maturity. Males were also younger than females at sexual maturity. A head start on growth by males may underlie their earlier maturation at a smaller size, thus leading to female-biased SSD at the adult stage.

## Introduction

Sexual size dimorphism (SSD) is a common feature of animals, and understanding the adaptive significance, developmental basis, and genetic architecture underlying diverse patterns of SSD has been a major focus of evolutionary research [1]. For example, there is interest in whether sex-specific differences during development represent a correlated response to sexual selection acting on adults or different optima for males and females [2,3]. In adult Pacific salmon in the genus *Oncorhynchus*, both male-biased and female-biased SSD is represented, depending on the species and population [4,5]. Among species, SSD ranges from male-biased values in which males are 4% larger than females (in chum salmon *O. keta* [Walbaum in Artedi, 1792]) to female-biased values in which males are 9% smaller than females (in Chinook salmon *O. tshawytscha* [Walbaum in Artedi, 1792])[4]. SSD may result from differences in the ages at which males and females are sampled or from sex differences in behaviour and development – differences that may begin to accumulate early in development. The current study focused on the unresolved question of whether SSD is present very early in development, when juveniles are still relying on yolk reserves. 

One factor that limits how early SSD can be measured is simply the stage at which individual fish can be sexed from the morphology of gonads. Based on histological examination, gonads begin to visibly differentiate late in the alevin stage (approximately 30 days post hatching), roughly coinciding with the emergence of free-swimming alevins from the incubation substrate, and the concomitant initiation of exogenous feeding (e.g., [6]). Given this limit on when gonads can be differentiated, all studies of SSD to date have been limited to post-emergent fry or even older life history stages (e.g., [7,8]). 

With the development of molecular markers linked to the Y chromosome, it is now possible to determine the sex of individual Pacific salmon prior to gonad differentiation [9,10,11,12]. In some cases, the correspondence between genetic sex and phenotypic sex is not perfect due to the presence of XY females. However, in an analysis of 55 *O. tshawytscha* populations, only 2% of the 1133 genetic males (*OtY1*M or *OtY1*MF) were phenotypic females [11]. With such a close correspondence between genetic sex and phenotypic sex, sexing by molecular markers appears to be a robust method for *O. tshawytscha*. In this study, our goal was to use a Y-linked genetic marker to sex morphologically-undifferentiated, pre-emergent *O. tshawytscha* alevins, and test for SSD by comparing the body size of male and female siblings. 

 SSD at the juvenile stage is often considered to reflect a correlated response to selection on adults. At the adult stage, patterns of SSD relate to differences in the strength of natural and sexual selection on males versus females [1]. In *O. tshawytscha*, most populations express female-biased SSD at the adult stage, with male-biased SSD only among the largest-bodied populations (e.g., mean male length > 80 cm)[4]. This pattern is consistent with Rensch’s Rule, a pattern in which male-biased SSD increases with body size in species in which males are generally larger than females, but decreases with body size in taxa in which females are generally larger than males. If selection on growth rate is correlated between juveniles and adults (the ‘correlated selection’ hypothesis), we predict female-biased SSD at the juvenile stage. On the other hand, a younger age at maturity in males than females may be the mechanism underlying female-biased SSD at the adult stage. Under this ‘life history’ hypothesis, we predict male-biased SSD at the juvenile stage, because faster juvenile growth is associated with an earlier age at maturity in general [13,14] and in *O. tshawytscha* [15]. Our experiment permitted a robust test of these competing hypotheses, and with evidence of male-biased SSD, our results are more consistent with the life history hypothesis.

## Methods

### Ethics Statement

All procedures on adult fish were approved by Western University’s Animal Use Subcommittee under Animal Use Protocol 2008-077 in accordance with The Canadian Council on Animal Care. Discomfort to animals was minimized by quickly transferring captured fish to the anesthetic bath, ensuring anesthetic induction was reached within 3 minutes, completing procedures (for this and other studies) as quickly as possible, and ensuring recovery prior to release. The capture of fish and the collection of gametes were approved by the Ontario Ministry of Natural Resources. All procedures on juveniles were approved by Western University’s Animal Use Subcommittee under Animal Use Protocol 2007-043 in accordance with The Canadian Council on Animal Care. Discomfort to animals was minimized by following high standards for husbandry and by applying the proper anesthetic dose for euthanasia.

### Study population

 Our study focused on an introduced *O. tshawytscha* population spawning in the Sydenham River, Owen Sound, Ontario, Canada (44°32.7’N, 80°56.2’W). The ancestral population from the Green River, Washington, USA [16] has an ‘ocean-type’ life history [17], in which juveniles migrate to the ocean in their first summer [18]. To the best of our knowledge, this life history has persisted in our population – except age 0 fish migrate to the Georgian Bay and Lake Huron – and no individuals become stream residents. Based on in-river sampling of sexually-maturing adults for a different study in 2011, females are larger than males (fork length = 75.4 ± SE = 0.6 cm [n = 112] versus 69.8 ± 0.7 cm [216], respectively). This SSD is largely attributable to many fewer females than males with fork lengths in the 40-70 cm range (17% versus 83%). Data from 2010 also indicate an older mean age of maturity in females than in males (4.2 ± 0.1 years [22] versus 3.6 ± 0.2 years [27]). 

### Breeding and Experimental Design

Sexually mature *O. tshawytscha* (10 females and 6 males) were captured between 29 September - 4 October 2011 using dip nets at the Mill Dam Fishway on the Sydenham River. Fish were anaesthetized with clove oil (20 mg·l^-1^ of water), the vent area was wiped dry with paper towel, and gametes were extracted by gently compressing the abdomen. Fish were then recovered in fresh water and released. Gametes were stored in a conventional cooler at approximately 4 °C, and were transported to Western University within 24 h. The diameters of 30 eggs per female were measured to estimate mean egg diameter. 

To the extent possible, we followed a nested half-sib-full-sib breeding design (i.e., North Carolina I) to control for the effects of sire (sire-effect variance) and dam (which includes the dam-effect variance and the dam × sire interaction variance)[19]. Whereas sire effects reflect the genetic contribution to phenotypic variation, dam effects reflect genetic and maternal contributions. Because gametes were collected over multiple days, we could not use a fully balanced North Carolina I design or a full factorial North Carolina II design. The final parental combinations were one dam per sire (*n* = 3 dams, *n* = 3 sires), two dams per sire (*n* = 4 dams, *n* = 2 sires), and three dams per sire (*n* = 3 dams, *n* = 1 sire). The eggs were fertilized and incubated following Ontario Ministry of Natural Resources best management practices (Fish Culture Technical Bulletin 2011-01, Ontario Ministry of Natural Resources, P.O. Box 7000, 300 Water Street, Peterborough, Ontario, Canada). In each cross, the male was used to produce four replicate families of 30 eggs each. Fertilized eggs were transferred to PVC cups (H = 50 mm, D = 50 mm) that had 2 mm mesh (1.5 x 2.1 mm) bottoms and drill holes (to increase porosity). Eggs were then disinfected in a dilute Ovadine® (Syndel Laboratories Ltd., Qualicum Beach, British Columbia, Canada) bath (50 ml·l^-1^of water) for 30 minutes. Cups were then transferred to a 10 °C water bath to allow the eggs to water harden for 1 h. 

Replicate families were reared at 10 °C and 15 °C in separate eight-tray vertical incubators (MariSource, http://www.marisource.com/) that were plumbed with dechlorinated, oxygenated, and filtered city water at a flow rate of 4-5 l·min^-1^ and a recirculation rate >95%. Over the course of the experiment, the mean temperature of each system was 9.8 ± SD = 0.8 °C and 15.3 ± 0.04 °C. A temperature of 10 °C was used because it is in the middle range of temperatures resulting in peak survival of embryos and alevins [20]. A temperature of 15 °C was used in case sex differences became more apparent when development was accelerated. In each stack, all experiment cups were kept on a single tray. Separate analyses were done for each temperature, and replicate families were pooled.

Alevins were allowed to develop for 35 days post hatch (dph) at 10 °C or 14 dph at 15°C. These periods corresponded to 90% of the time between hatch and the expected time to reach maximum alevin wet weight (MAWW; Table 5 in [21]). It was assumed that sex differences in wet weight (i.e., within families) would reflect sex differences in yolk absorption rate and/or yolk conversion efficiency. Euthanasia occurred by immersing alevins for 10 minutes in a solution of tricaine methane sulfonate (300 mg·l^-1^ of water) buffered with sodium bicarbonate (300 mg·l^-1^ of water). Each alevin was then measured for standard length with dial calipers and weight (*W*) using a digital balance (± 0.1 mg). The tail of each alevin was clipped and stored individually in 95% ethanol. The number of alevins that survived to the endpoint to be measured and sexed was 461 (25-57 per maternal family) in the 10 °C treatment and 403 (n = 27-56 per maternal family) in the 15 °C treatment.

### DNA Analysis

DNA was extracted from tail clippings using a standard Chelex extraction [22]. The alevins were then genotyped at the *OtY1* marker using PCR amplification and separation on an agarose gel as described in Devlin et al. (1994). The banding pattern for this *OtY1* PCR-based test clearly distinguished genetic males and females, with males showing a single bright band at approximately 200 bp and females showing multiple dull bands longer than 200 bp [9]. 

### Statistical Analysis

 Linear mixed models were used to test for the effects of sex on *W* while taking into account differences among sires and differences among dams nested within sires using a standard animal model approach [23,24]. The mixed model approach was able to handle the unbalanced design and permitted an individual-level (i.e., within families) analysis rather than a family-level (i.e., among families) analysis of SSD. In the family-level analysis, the unit of replication would be the family and the dependent variable would be the slope of the relationship between male size and female size. Although this analytical approach is typical in studies of SSD [1], it results in loss of statistical power and loss of information [25]. To justify the inclusion of parental effects, an unconditional means model was fit initially using the function lmer in lme4 in R version 2.10.1 [26].. In this linear mixed model, sire and dam nested within sire were modeled as random effects. The intraclass correlation (ρ) was used to estimate how much of the variability in *W* was associated with differences among dams or sires:

$$\rho_{dam}=\frac{s_{dam}^{2}}{s_{dam}^{2}+s_{sire}^{2}+s_{wg}^{2}}$$

$$\rho_{sire}=\frac{s_{sire}^{2}}{s_{dam}^{2}+s_{sire}^{2}+s_{wg}^{2}}$$

Here, $s_{dam}^{2}$is the variance among dams, $s_{sire}^{2}$is the variance among sires, and $s_{wg}^{2}$ is the residual variance [25].

Sex and mean egg diameter were included in the animal model as fixed effects and were analyzed in a Bayesian framework using the R function MCMCglmm [23,27]. Sex was coded as a dummy variable (0 = male, 1 = female) and egg diameter was centered to have a mean of 0. If the egg diameter by sex interaction was non-significant, it was removed. Fixed effects were considered significant if the posterior distributions did not overlap zero. To assess the robustness of results for sex, linear mixed models were also implemented using the lme R function in the nlme package and using PROC MIXED in SAS. Similar results (not shown) were found using these different approaches. Analyses were also done using length as the dependent variable, but no sex differences were found (i.e, the posterior distribution overlapped zero; results not shown).

To evaluate the potential for false assignment of sex (or sex-biased mortality) at each temperature, the number of males and females was compared to an equal sex ratio using χ^2^ tests. In addition, the number of males and females was compared among maternal families using χ^2^ contingency tables. To evaluate whether the relationship between *W* and sex depended on mortality, the proportion of eggs that were fertilized and survived to 90% MAWW (centered to have a mean of 0) was included as a fixed effect in the mixed models, but it did not account for significant variation in *W* at either temperature (results not shown).

## Results

The mean proportion of eggs that were fertilized and survived to 90% MAWW was 0.77 per family at 10 °C and 0.67 per family at 15.3 °C. At 10 °C, the overall sex ratio of 52.3% males did not differ from 50% (χ^2^ test: χ^2^_1_=0.96, *P*=0.33) and was similar among families (χ^2^ test: χ^2^_9_=10.3, *P*=0.33). At 15 °C, the overall sex ratio of 49.4% did not differ from 50% (χ^2^ test: χ^2^_1_=0.06, *P*=0.80) and was similar among families (χ^2^ test: χ^2^_9_=7.7, *P*=0.57). 

In the unconditional means model at 10 °C, ρ_*dam*_ = 0.889 and ρ_*sire*_ = 0.040. Thus, 88.9% of the variability in *W* was associated with differences among dams and 4.0% of the variability in *W* was associated with differences among sires. In the linear mixed model with fixed effects, the interaction between sex and egg diameter was not significant (the posterior distribution overlapped zero) and so was removed. In the final model, *W* increased with egg diameter (posterior mode=108.2, 95% CI of posterior distribution=64.3-144.3) but did not differ between sexes (mode=0.71, CI=-3.03-3.04). Of the variation accounted for by differences among dams (88.9%), 86% was accounted for by differences in mean egg diameter.

**Figure 1:**
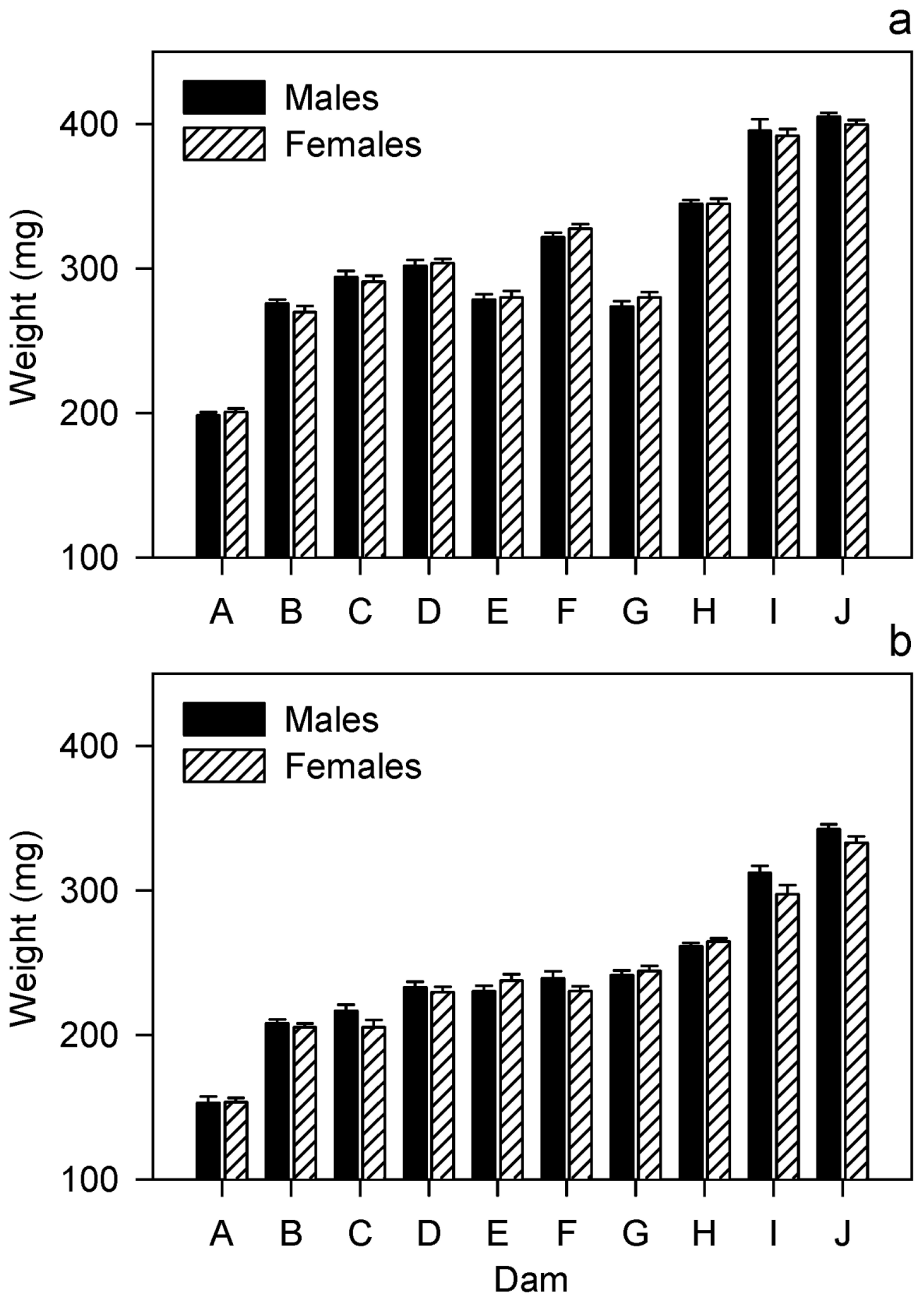
A comparison of weight between male and female alevins in Chinook salmon at 10 °C (a) and 15 °C (b). Alevin weight (mean ± SE) is shown for males and females from 10 dams (coded as capital letters). At 15 °C, sex accounted for 2% of the residual variance after controlling for the effects of sire (not shown), dam nested within sire, and mean egg diameter (not shown). Dams are sorted according to alevin weight at 15 °C.

In the unconditional means model at 15 °C, ρ_*dam*_ = 0.681 and ρ_*sire*_ = 0.216. Thus, 68.1% of the variability in *W* was associated with differences among dams and 21.6% of the variability in *W* was associated with differences among sires. In the linear mixed model with fixed effects, *W* increased with egg diameter (mode=76.1, CI=37.1-111.6) and differed between sexes (Figure 1). On average, males were 3.67 mg (CI=0.30-7.0) larger than their female siblings. In addition, the effect of sex depended on egg diameter (mode=8.9, CI=1.6-16.1), with the effect of sex being larger in families with larger eggs. Of the variation accounted for by differences among dams (68.1%), 86% was accounted for by differences in mean egg diameter. Of the residual variation present in the unconditional case (*s*_*wg*_^2^ = 301.0), the inclusion of sex accounted for 2% of the variance.

## Discussion

There was a statistically significant but small degree of sexual dimorphism at 15 °C, with males being 2% heavier than females. Such male-biased SSD directly opposes the correlated selection hypothesis but is consistent with the life history hypothesis for SSD in *O. tshawytscha*. Similar to other populations of *O. tshawytscha* in which body lengths are < 80 cm, we observed female-biased SSD at sexual maturity in the Sydenham River population. We also observed a younger age at maturity in males than in females, and earlier maturity is usually associated with faster growth. We suggest that a head start on growth by males underlies their earlier maturation, and thus contributes towards female-biased SSD at sexual maturity.

Detecting male-biased SSD at the late alevin stage was possible because of careful control of temperature and family effects, an analysis that focused on individual- not family-level variation, and possibly, the acceleration of development at 15°C that may have exaggerated SSD. In addition, SSD was more apparent in families from larger eggs. The physiological mechanisms underlying the observed SSD within families are not yet understood but must relate to how endogenous nutrient reserves (i.e., yolk) are metabolized, because alevins were not yet free-feeding. In addition, different hormone profiles, evident already at hatching [6], may be involved in initiating a cascade of changes that eventually result in SSD.

At 90% MAWW, the sex ratio did not deviate from 50:50. This is consistent with a primary sex ratio of 50:50 caused by the XY sex determining mechanism in *O. tshawytscha* and a close 1:1 correspondence between genetic sex and phenotypic sex [11]. It is also consistent with comparable embryo mortality between the sexes. Thus, the test of SSD in this study did not appear to be influenced by sex-biased mortality. Furthermore, SSD was not related to family differences in the proportion of eggs that were fertilized and survived until 90% MAWW. 

Similar to the current study, *O. masou* (Brevoort, 1856) reared for 60 days after emergence also showed male-biased SSD, with SSD only apparent if alevins had hatched from relatively large eggs [8]. However, other studies have failed to detect sexual dimorphism in behaviour, growth, or size at early life history stages (e.g., [28,29]). Whereas different degrees and forms of SSD appears to be common at sexual maturity [4,5], it may be variable in its expression during development or simply may be too small to be easily detected at early life history stages. It seems likely that the form and degree of SSD varies across life history stages.

 On the one hand, a small degree of SSD at the late alevin stage could have ecological significance. Yamamoto [8] suggested that males were larger than females at 60 days post emergence because males were able to secure better foraging territories than females. Sexual size dimorphism during the alevin stage may benefit males during territory acquisition for several reasons. First, SSD may carry over to emergence, thus giving males a size advantage during competition for territories. Second, larger individuals (i.e., males) may emerge earlier [30] and thus may acquire a prior resident advantage. Third, large size is associated with a higher metabolic rate, which also can improve social status [31]. On the other hand, the amount of SSD was small relative to the large size differences among families and SSD was only evident at the higher temperature. 

Our results have implications for experimental design in studies of early life history in salmonid fishes. Given the small degree of SSD observed in our study, it should not be necessary to control for sex in studies at the pre-feeding stage. However, it is still important to account for parental effects, as also advocated by Burt et al. [32]. In our study, most of the variation in *O. tshawytscha* alevin weight (*W*) was attributed to differences among dams (i.e., 88.9% at 10 °C; 68.1% at 15 °C), with most (86%) of the explained variation among dams due to variation in mean egg diameter. This strong maternal effect on *W* is consistent with other studies of *Oncorhynchus* spp. [20] Sire accounted for much less of the variation in alevin *W* (0.0% at 10 °C; 21.6% at 15 °C). Sire effects typically start weak and strengthen with developmental age as maternal effects weaken [33]. 

Female-biased SSD is the typical pattern observed at sexual maturity in *O. tshawytscha*. We considered two competing hypotheses for the developmental basis of female-biased SSD and developed predictions for SSD at the juvenile stage. The correlated selection hypothesis, in which selection at one life history stage depends on selection at another life history stage, was too simplistic, in part because it assumes a similar age at maturity between males and females. In contrast, the life history hypothesis was better because it allows for differences in age at maturity between males and females. However, neither hypothesis considers sex-specific optima for body size or growth at the juvenile stage. Future research is needed to explore inter-dependencies between stage-specific selection, and their role in the evolution and ecology of SSD. 
